# Myocardial imaging with 18F-fluoro-2-deoxyglucose positron emission tomography and T2-weighted short tau inversion recovery black-blood images of cardiac magnetic resonance in cardiac sarcoidosis

**DOI:** 10.1186/1532-429X-18-S1-P253

**Published:** 2016-01-27

**Authors:** Reina Tonegawa, Takatomo Nakajima, Hirotake Takahashi, Mitsuyoshi Mita, Keisuke Shirasaki, Shinya Fujii, Akimichi Murakami, Takeyukii Kubota, Teruhiko Suzuki, Yosuke Nakano, Kensuke Fujiwara, Takashi Miyamoto, Makoto Mutoh

**Affiliations:** Department of Cardiology, Saitama Cardiology&Respiratory Center, Saitama, Japan

## Background

Cardiac sarcoidosis (CS) may be life threatening because of end-stage cardiomyopathy and sudden cardiac death. The frequent absence of specific symptoms and lack of a diagnostic method pose challenges in CS diagnosis. Endomyocardial biopsy is invasive and has low diagnostic sensitivity due to sampling errors. Thus, non-invasive cardiac imaging has an important role in detecting cardiac involvement of sarcoidosis. Recently, cardiac magnetic resonance (CMR) and 18F-fluoro-2-deoxyglucose positron emission tomography (18F-FDG-PET) have been reported as a promising diagnostic modality in identifying cardiac involvement of sarcoidosis. Late gadolinium enhancement (LGE) of CMR typically reveals areas of irreversible damage, such as fibrosis, whereas T2-weighted short tau inversion recovery black-blood image (T2-STIR BB) reveals edematous tissue; 18F-FDG-PET reveals myocardial inflammation. Particularly, the latter two modalities are possibly useful in assessing the necessity or dose of steroid therapy. We compared T2-STIR and 18F-FDG PET results in patients with suspected cardiac involvement.

## Methods

We used "Japanese Ministry of Health and Welfare (JMHW) guidelines for the diagnosis of CS" for this study. Patients who met more than 2 major criteria, or 1major and 2 minor criteria are defined as "definite" CS patients. Those who met at least 1 major or minor criteria, but not definite CS, are defined as "suspected" CS patients. Twenty consecutive definite or suspected CS patients underwent CMR and 18F-FDG-PET. The T2-STIR BB, LGE, and 18F-FDG PET data was interpreted by experienced physicians. All subjects underwent evaluation of LV function and structure using echocardiography.

## Results

Nine of the 20 patients were definite CS patients, whereas the other eleven were suspected CS patients. The sensitivity and specificity for diagnosing definite CS were 88.9% and 54.5%, respectively, for 18F-FDG-PET and 55.6% and 18.2%, respectively, for T2-STIR BB. All subjects had patchy or non-vascular distributed LGE pattern. When the 18F-FDG-PET and T2-weighted MRI images were compared, 18/20 patients tested positive by one (n = 9) or both (n = 9) of the two modalities. Of the nine patients testing positive by only one modality, five were positive by only T2-STIR BB and four by only 18F-FDG-PET. Two other patients tested negative by both image modalities but had clear involvement of the lungs and thus were suspected to have had CS in the past, although not currently active.

## Conclusions

18F-FDG PET provided high sensitivity for diagnosing CS in patients with suspected cardiac involvement. The specificity and sensitivity of T2-STIR BB were lower than those of 18F-FDG PET. Results from T2-STIR BB and 18F-FDG PET did not correspond in 45% of the cases.

